# Hypocaloric diet supplemented with probiotic cheese improves body mass index and blood pressure indices of obese hypertensive patients - a randomized double-blind placebo-controlled pilot study

**DOI:** 10.1186/1475-2891-12-138

**Published:** 2013-10-12

**Authors:** Khaider K Sharafedtinov, Oksana A Plotnikova, Ravilay I Alexeeva, Tatjana B Sentsova, Epp Songisepp, Jelena Stsepetova, Imbi Smidt, Marika Mikelsaar

**Affiliations:** 1 Federal State Budgetary Institution “Institute of Nutrition” under the Russian Academy of Medical Sciences, 2/14 Ustinsky proezd, Moscow 115446, Russian Federation; 2 State Budgetary institution of Continuing Professional Education “Russian Medical Academy of Postgraduate Education”, St. Petersburg 191015, Russian Federation; 3 Bio-Competence Centre of Healthy Dairy Products LLC, Kreutzwaldi str. 1, Tartu 51014, Estonia; 4 Department of Microbiology, University of Tartu, Ravila str. 19, Tartu 50411, Estonia

**Keywords:** Obesity, Hypocaloric diet, Probiotic cheese, *Lactobacillus plantarum* TENSIA, Cholesterol, Plasma glucose, Plasma lipids, Blood pressure, Body composition, Urine polyamines, Fecal Lactobacilli

## Abstract

**Background:**

Gut lactobacilli can affect the metabolic functions of healthy humans. We tested whether a 1500 kcal/d diet supplemented with cheese containing the probiotic *Lactobacillus plantarum* TENSIA (Deutsche Sammlung für Mikroorganismen, DSM 21380) could reduce some symptoms of metabolic syndrome in Russian adults with obesity and hypertension.

**Methods:**

In this 3-week, randomized, double-blind, placebo-controlled, parallel pilot study, 25 subjects ingested probiotic cheese and 15 ingested control cheese. Fifty grams of each cheese provided 175 kcal of energy. Blood pressure (BP), anthropometric characteristics, markers of liver and kidney function, metabolic indices (plasma glucose, lipids, and cholesterol), and urine polyamines were measured. Counts of fecal lactobacilli and *L*. *plantarum* TENSIA were evaluated using molecular methods. The data were analyzed by t-test for independent samples and Spearman’s partial correlation analysis.

**Results:**

The probiotic *L. plantarum* TENSIA was present in variable amounts (529.6 ± 232.5 gene copies) in 16/25 (64%) study subjects. Body mass index (BMI) was significantly reduced (p = 0.031) in the probiotic cheese group versus the control cheese group. The changes in BMI were closely associated with the water content of the body (r = 0.570, p = 0.0007) when adjusted for sex and age. Higher values of intestinal lactobacilli after probiotic cheese consumption were associated with higher BMI (r = 0.383, p = 0.0305) and urinary putrescine content (r = 0.475, p = 0.006). In patients simultaneously treated with BP-lowering drugs, similar reductions of BP were observed in both groups. A positive association was detected between TENSIA colonization and the extent of change of morning diastolic BP (r = 0.617, p = 0.0248) and a trend toward lower values of morning systolic BP (r = −0.527, p = 0.0640) at the end of the study after adjusting for BMI, age, and sex.

**Conclusion:**

In a pilot study of obese hypertensive patients, a hypocaloric diet supplemented with a probiotic cheese helps to reduce BMI and arterial BP values, recognized symptoms of metabolic syndrome.

**Trial registration:**

Current Controlled Trials ISRCTN76271778

## Introduction

Obesity, obesity-related disorders, and metabolic syndrome have become an epidemic in Western societies. Obesity results from complex interactions between genes and environmental factors such as diet, food components, and lifestyle. Metabolic syndrome consists of a group of factors involved in an increased risk of developing cardiovascular diseases and type 2 diabetes. Three or more of the following signs define metabolic syndrome: obesity and insulin resistance, increased blood pressure (BP), high fasting blood triglycerides and glucose, and low high-density lipoprotein levels [1,2]. Alvarez-Leon et al. [3] have pointed on the inverse association between ingestion of dairy products and high BP. Low-fat spreads containing bioactive milk peptides were able to reduce systolic blood pressure (SBP) and serum cholesterol in hypertensive and hyper-cholesterolemic subjects [4]. However, the beneficial influence of dairy products on BP and cardiovascular health has not been assessed regarding cheese or other traditionally high-fat products [5].

Interactions between intestinal microbiota and host play an important role in the physiological regulation of metabolic functions and the development of various diseases. Different health-improving effects of various *Lactobacillus* spp. have been demonstrated after their application as natural or designer probiotics [6,7]. Probiotics are defined as live microorganisms that confer a health benefit to the host when administered in adequate amounts [8]. Probiotic *Lactobacillus* strains possess various functional properties for health promotion, including high antimicrobial activity against pathogens, cholesterol-lowering effects, antioxidative properties, and immunogenic potential [9-11]. *Lactobacillus helveticus*-fermented milk containing bioactive peptides reduced BP in hypertensive subjects when ingested daily [12]. However, strain-specific health effects may be associated with significant differences in the production of specific metabolites among the *Lactobacillus* strains [13-16].

Recent assessments of diets combined with probiotics have been directed towards the control of biomarkers of the host’s basic metabolism, particularly carbohydrates, lipids, and amino acid turnover after dairy probiotic administration for different hosts [17,18]. However, whether the addition of a probiotic strain to full-fat dairy products can improve the functionality indices of the host remains to be elucidated.

This study evaluates the clinical efficacy of a hypocaloric diet supplemented with cheese with a moderate fat content that includes the probiotic *Lactobacillus plantarum* TENSIA (Deutsche Sammlung für Mikroorganismen, DSM 21380) in Russian adult patients with obesity and hypertension with particular accompanying diseases under standard treatment. BP, anthropometric characteristics, markers of liver and kidney function, metabolic indices (plasma glucose, lipids, and cholesterol), and urine polyamines were tested. Counts of fecal lactobacilli and intestinal *L*. *plantarum* TENSIA survival were evaluated using molecular methods.

## Materials and methods

### Probiotic *Lactobacillus* strain

*L. plantarum* TENSIA was previously isolated from the gastrointestinal tract of healthy Estonian children [19]. The strain *L. plantarum* TENSIA® has been deposited in the Deutsche Sammlung von Mikroorganismen und Zellkulturen [German Collection of Microorganisms and Cell Cultures] GmbH under registration number DSM 21380 at 16.04.2008. Molecular identification of the strain as *L. plantarum* was confirmed by internally transcribed spacer polymerase chain reaction and 16SrRNA sequencing [20]. The safety of the strain was confirmed previously using an animal model [21], as well as in Estonian healthy adults and elderly subjects (registered trials ISRCTN38739209 and ISRCTN45791894) [22].

### Cheese preparation

The probiotic cheese containing *L. plantarum* TENSIA was developed at E-Piim Production in Estonia under the trademark Harmony™ [22]. Two similar cheeses were prepared on the basis of regular Edam-type cheese with a starter C92 (CSK Food Enrichment,Netherlands). To produce the probiotic cheese, the *L. plantarum* TENSIA was added to the cheese milk in amounts of 1.5x10^11^ CFU/g before renneting. Both cheeses (probiotic and control) were similarly ripened for 4 weeks at 10-12°C and 80-85% relative air humidity. The microbial composition of test and control cheeses did not differ in the counts and prevalence of non-starter microbiota. In probiotic cheese the viability of *L. plantarum* TENSIA was assessed before the human trial. The energy provided by consumption of 50 g of each cheese was 175 kcal.

The composition and energy value (1512 kcal) of a standard hypocaloric diet is depicted in Table 1.

**Table 1:** Composition and energy value of a standard hypocaloric diet

**Substrate**	**Value**
Energy value, kcal	1512
Protein, g (%)	81 (21.4)
Vegetable protein, g	28.3
Fat, g (%)	52 (31.0)
Vegetable fat, g	23.4
Saturated fatty acids, g (%)	14.4 (8.5)
Monounsaturated fatty acids, g (%)	16.5 (9.7)
Polyunsaturated fatty acids, g (%)	15.3 (9.0)
Cholesterol, mg	231
Carbohydrates, g (%)	180 (47.6)
Fiber, g	24.6
Hemicellulose	10.0
Cellulose	8.6
Pectin	6.0
Vitamins, mg	
Ascorbic acid	102.3
Thiamin B-1	0.53
Riboflavin B-2	1.13
Pyridoxine B-6	1.47
Niacin	11.6
Vitamin А	0.3
Beta-carotene	3.64
Vitamin E	11.3
Minerals,mg	
Potassium	2685
Calcium	1007
Magnesium	342
Sodium	2124
Phosphorus	1154
Iron	11.4
Copper	2.16
Zinc	12.4
Chromium	0.18
Manganese	4.63
Iodine	0.14
Probiotic count in cheese, log CFU/g	8.7
Daily dose of probiotic, logCFU	10.4
Daily dose of cheese, g	50
Fat intake with cheese, g/d	13
Saturated fat intake with cheese, g/d	8
Cholesterol intake with cheese, mg/d	45

### Study population

Participants in the clinical trial were randomly selected from consecutive patients admitted to the Clinic of the Institute of Nutrition (Russian Academy of Medical Sciences) from January 2011 until March 2011. The number of expected hospitalized patients (approximately 40 patients for 3 months) relevant to inclusion criteria was postulated. Inclusion criteria for the patients were: age 30–69 years, a diagnosis of metabolic syndrome characterized by obesity accompanied by arterial hypertonia (>130/85 mm Hg), the absence of uncompensated chronic diseases needing intensive treatment, and informed consent for participation and treatment with a hypocaloric diet supplemented with a probiotic. Exclusion criteria included a history of gastrointestinal disease, food allergy or acute infection, use of any antimicrobial agent within the preceding month, pregnancy or breast-feeding, and no wish to participate. The primary outcomes were defined both as a significant (p < 0.05) decrease of arterial blood pressure and body mass index (BMI).

All participants provided written informed consent and were informed that they could withdraw from the study at any time; however, no withdrawals were registered during the hospital stay period.

Study participants were randomly allocated according to SPSS 17 for Windows into two groups.

**Figure 1:**
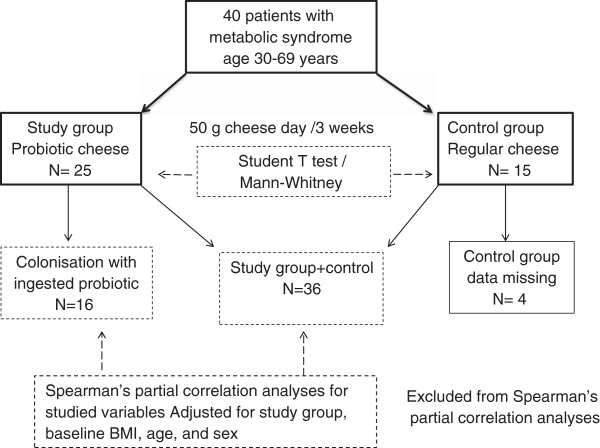
**Patient dispensation throughout the study and the statistical analysis.** Blocked randomization lists were produced by the statistician (MV) and held centrally. All invited hospitalized patients attended the study and gave written informed consent before inclusion.

The 40 patients were randomly divided into treatment and control groups according to the mode: 2 patients of treatment group vs.1 patient for control group (Figure 1). The randomized, double-blind, placebo-controlled, parallel-designed, two-armed intervention study was performed and conducted according to the guidelines laid down in the Declaration of Helsinki 1996–2000. The Ethical Committee of the Institute of Nutrition of AMS of Russia approved the study protocol. The trial was completed in accordance with good clinical practice [registered trial ISRCTN76271778]. The demographic, clinical, and medication data of the study and control groups are presented in Table 2.

**Table 2:** Demographic, clinical, and medication data of patients in probiotic and control groups

**Characteristics**	**%**	**Control group N = 15**
Male, n (%)	9 (36%)	4 (27%)
Female, n (%)	16 (64%)	11 (73%)
Age, years	52.0 ± 10.9	51.7 ± 12.1
Body weight, kg	105.6 ± 16.2	102.4 ± 14.4
Body mass index, kg/m^2^	37.7 ± 4.3	36.3 ± 4.1
Stage I, n	4	3
Stage II, n	12	8
Stage III, n	9	4
Systolic BP, mm Hg	134.0 ± 8.0	131.4 ± 6.6
Stage I, n	2	3
Stage II, n	16	8
Stage III, n	7	4
Diastolic BP, mm Hg	82.4 ± 6.1	82.1 ± 5.8
Drugs used, n (%)	21 (84%)	12 (80%)
Ca antagonist	4	2
Beta-blocker	4	3
ACE inhibitor	12	7
Diuretics	2	1
Others	2	1
Smokers, n (%)	3	2

Data are presented as mean ± standard deviation unless otherwise noted. Fisher’s exact test and *t*-test: There were no statistical differences between any indices in the two patient groups. BMI scale: overweight = BMI of 25–29.9 kg/m^2^; grade 2 overweight (commonly called obesity) = BMI of 30–39.9 kg/m^2^; grade 3 overweight (commonly called severe or morbid obesity) = BMI ≥40 kg/m^2^. (NORIP, http://www.furst.no/norip/). Blood pressure (BP) scale: Grade 1 hypertension = systolic blood pressure (SBP) 140–159 and/or diastolic blood pressure (DBP) 90–99; Grade 2 hypertension = SBP 160–179 and/or DBP 100–109; Grade 3 hypertension = SBP ≥180 and/or DBP ≥110 (28).

### Study protocol

Altogether, the trial lasted for 3 weeks. For the treatment group, the standard hypocaloric diet was supplemented with 50 g/day of probiotic product (semi-hard cheese) containing *L. plantarum* TENSIA. Members of the control group consumed the same amount of a probiotic-free cheese (Table 1). Implementation and allocation methods were determined by the clinical coordinator (KS, Clinic of Institute of Nutrition) during the participants’ stay in the hospital. The technical assistant confirmed the consumption of the cheese, and was blinded to which cheese contained or lacked probiotic. The medical doctors keeping the records were also blinded to cheese type. The code was kept in sealed envelopes and opened after all results of clinical and laboratory analyses had been registered.

Anthropometric markers were measured, and the effect of the diet supplemented with the probiotic cheese on patients’ health indices was evaluated. The body composition, e.g. fat and muscle mass and total body water content were estimated by bioelectrical impedance analysis (InBody 720, Korea). BP was measured twice daily (morning and evening) at the hospital; BP was measured on the right arm using a sphygmomanometer, with subjects in sitting position after 5 min rest. After BP was measured, blood samples were collected at recruitment (day 0) and at the end of the trial (day 21). Plasma glucose, plasma lipids, cholesterol, and markers of liver and kidney function [aspartate aminotransferase (AST), alanine aminotransferase (ALT), and serum creatinine] were determined with standard laboratory methods using certified assays in the clinical laboratory of the hospital (Table 3). Intervals for routine laboratory tests proposed by the Nordic Reference Interval Project (NORIP) were used as references [23].

**Table 3:** Laboratory values after treatment

**Laboratory measurement**	**Reference value**	**Probiotic cheese**			**Control cheese**			**Extent of changes**
		**Baseline**	**After treatment**	***P***	**Baseline**	**After treatment**	***P***	**Probiotic vs. Control** ***P***
Total cholesterol, mmol/L	<5.2	5.32 ± 1 25	4.09 ± 1.20	<0.001	5.34 ± 0.95	4.35 ± 0.87	0.004	0.495
LDL, mmol/L	< 3.8	3.08 ± 1.11	2.20 ± 0.92	0.004	3.21 ± 0.69	2.64 ± 0.66	0.021	0.276
HDL, mmol/L	< 1.15	1.13 ± 0.25	0.94 ± 0.17	0.006	1.14 ± 0.26	1.05 ± 0.22	0.229	0.128
TG, mmol/L	< 1.7	2.80 ± 2.01	2.09 ± 1.62	0.041	2.14 ± 1.16	1.43 ± 0.56	0.085	0.696
Serum glucose, mmol/L	3.9 -5.8	7.16 ± 2.84	5.87 ± 1.00	0.171	6.84 ± 1.92	5.64 ± 1.6	0.014	0.875
Serum creatinine, μmol/L	50 – 105	80.8 ± 20.0	69.2 ± 19.4	0.003	70.5 ± 13.6	57.3 ± 7.4	0.007	0.394
Urea, μmol/L	2.5 – 6.4	4.72 ± 1.21	5.62 ± 2.29	0.017	4.66 ± 2.71	3.89 ± 0.98	0.245	0.011
Uric acid, μmol/L	200 – 340	356.1 ± 85.7	393.4 ± 128.6	<0.07	326.7 ± 147.7	308.8 ± 111.4	0.49	0.252
Total bilirubin, μmol/L	< 20	14.9 ± 5.68	12.6 ± 4.24	0.003	14.2 ± 4.83	11.3 ± 3.61	0.005	0.547
AST, U/L	0 – 35	23.5 ± 8.02	27.8 ± 9.86	<0.053	31.5 ± 16.6	25.0 ± 10.5	0.084	0.468
ALT, U/L	0 – 35	32.8 ± 16.0	36.3 ± 20.9	0.390	45.3 ± 36.4	35.9 ± 26.1	0.149	0.118

ALT, alanine aminotransferase; AST, aspartate aminotransferase; HDL, high-density lipoprotein;

LDL, low-density lipoprotein; TG, triglycerides.

Urine and fecal samples were collected before and after completing the trial and sent to Estonia, University of Tartu, Biomedicum. The urinary creatinine and polyamine content was tested. In fecal samples, the survival of *L. plantarum* TENSIA and the number of total lactobacilli were estimated.

### Detection of polyamines in cheese and urine samples

GC analysis was performed using a Hewlett-Packard HP model 6890 gas chromatograph (Hewlett Packard, USA) equipped with a split/splitless capillary inlet system and a flame ionization detector. The GC column was a 30 m × 0.32 mm (i.d.) fused silica capillary coated with cross-linked 5% phenylmethyl silicone (film thickness 0.25 mm). The detector temperature was 350°C and the injector temperature was 200°C. The oven temperature program was a gradient system: the initial temperature was 150°C, it increased to 280°C at a rate of 20°C/min, and it remained at 280°C for 4.5 min. The chromatographic peak areas were integrated with a Hewlett-Packard networking integrator.

#### Cheese

Ten grams of cheese were weighed, chopped into very small fragments, and placed in a plastic bottle that contained 20 mL of a 50% methanol solution (HPLC grade, Aldrich, USA). The solution was homogenized by vortex mixing for 5 min. The cheese mix was then incubated at 45°C for 1 h. Next, the extract was cooled to 30°C and centrifuged at 4000 rpm for 15 min [24]. To detect poly- and biogenic amines, 200 μL of the supernatant was analyzed using propyl chloroformate (HPLC grade, Aldrich, USA). Polyamine concentrations in cheese were expressed in mg/L.

#### Urine

Urine samples (1 mL each) were mixed with 1 mL of dichloromethane containing 5 μL propyl chloroformate. After the first mixing, the pH of the mixture was increased to greater than 12 with 5 M sodium hydroxide solution. The aqueous phase was removed; the organic phase was centrifuged at 3000 rpm for 5 min, and the remaining aqueous phase was removed. Subsequently, the organic phase was concentrated under a stream of nitrogen, and 1 μL was injected onto the GC system. The calibration graphs were prepared using polyamine standard solutions at different concentrations (3–150 nmol); 1.6-diaminohexane was used as an internal standard. Polyamine concentrations in urine were expressed as mmol/mol of creatinine. Creatinine was measured calorimetrically using the Jaffe kinetic method [25].

### Molecular assessment of total lactobacilli and *L. plantarum* TENSIA

#### DNA extraction

Bacterial DNA from fecal samples was extracted using the QIA amp DNA stool mini kit (QIAgen, Hilden, Germany) with some modifications. First, 0.22 g of feces were re-suspended in 200 μL of TE buffer (10 mM Tris, 10 mM EDTA pH = 8, 20 mg/mL lysozyme, 200 u/mL mutanolysin) and incubated for 1 h at 37°C. Next, 0.3 g of 0.1 mm zirconia/ silica beads and 1.4 mL of ASL solution from the stool mini kit were added to the fecal samples. The tubes were then agitated for 3 min at 5000 rpm in a mini-bead beater (Biospec Products Inc., USA).The protocol was then continued as described by the manufacturer (QIAgen, Germany).

#### Quantitative analysis by real-time polymerase chain reaction (RT-PCR)

To establish a quantitative assay, we cloned plasmids containing the amplified region of target bacteria using the pGEM-T vector system (Promega, Madison, WI). The PCR amplicons for *L. paracasei* and *L. plantarum* TENSIA were individually inserted into separate plasmid vectors; then, the recombinant vectors were transformed into chemically competent *E. coli*. Plasmids were purified by MaxiPrep (Qiagen). The purified plasmids were quantified by spectrophotometry (Quibit^TM^, Invitrogen) of multiple dilutions [26]. Quantification of target DNA was achieved using serial 10-fold dilutions from 10^2^ to 10^8^ plasmid copies of the previously quantified plasmid standards. Plasmid standards and samples were run in triplicate, and the average values were used to calculate the bacterial load.

RT-PCR was performed using the ABI PRISM 7500 HT Sequence Detection System (Applied Biosystems, USA) with optical grade 96-well plates. The PCR reaction to detect total counts of lactobacilli was performed in a total volume of 25 μL using the SYBR Green PCR Master mix (PE Applied Biosystems, USA). Each reaction included 150 ng of template DNA, SYBR Green Mix (Applied Biosystems, USA), and a final concentration of 2 μM of each primer for total lactobacilli (Lac-F: 3’-AGCAGTAGGGAATCTTCCA-5’; Lac-R: 3’-CACCGCTACACATGGAG-5’)(27). The PCR conditions were as follows: 2 min at 50*°*C and 10 min at 95*°*C; followed by 40 cycles consisting of denaturation at 95°C for 15 s and annealing-elongation at 60*°*C for 1 min. To detect *L. plantarum* TENSIA, the reaction mixture (25 μL) for TaqMan assay contained 2×TaqMan Universal PCR Master Mix (PE Applied Biosystems, USA), 20 pmol of primers (Tens31: 3’-AACGCAAGCTTTATCCGATG-5’; Tens32: 3’-GTTAAGGTTTGCAACAGGTC-5’), 10 pmol of TaqMan probe (5’-FAM-ACCCGCGACGTACTTAAAAA-Tamra-3’), and 200 ng of extracted DNA. The thermocycling program was an initial cycleof 95*°*C for 10min, followed by 45 cycles of 95°C for 10 s and 60*°*C for 1 min. The negative controls were both PCR Master Mixes without DNA. Data analysis was conducted using Sequence Detection Software version 1.6.3, supplied by Applied Biosystems, USA.

### Statistical evaluation

The trial results were analyzed using SPSS 17 for Windows. Data were presented as mean *±* standard deviation or range with median, depending on the normal or non-parametric distribution of data. The *t*-test or Mann–Whitney rank sum test (numerical variables) and χ^2^test or Fisher’s exact test (categorical variables) were used to determine the between-group differences in smoking, drugs, and biological sex (Table 2). Differences from baseline values in treatment and control groups were evaluated using a paired *t*-test (normal distribution) or Mann–Whitney rank sum test (Tables 3, 4 and 5). Differences in the magnitude (extent) of change among tested indices (post-treatment values minus baseline values) between the treatment group and control group were evaluated using a *t*-test or Mann–Whitney test. Spearman’s partial correlation analysis, controlling the effects of group, baseline body mass index (BMI), age, and sex on different tested indices was applied. The correlation analysis was conducted in the SAS System® (SAS, Cary, NC, USA) using the CORR procedure (Table 6).

**Table 4:** Anthropometric indices and blood pressure values of patients after consumption of probioticor control cheese

**Index**	**Probiotic cheese**			**Control cheese**			**Extent of change**
	**Baseline**	**After treatment**	***P***	**Baseline**	**After treatment**	***P***	**Probiotic vs. control cheese*P***
Body weight, kg	105.6 ± 16.2	99.9 ± 14.4	<0.001	102.4 ± 14.4	98.0 ± 13.3	<0.001	0.083
BMI, kg/m^2^	37.7 ± 4.3	35.7 ± 3.8	<0.001	36.3 ± 4.3	34.7 ± 4.2	<0.001	0.031
Waist-to-hip ratio	0.985 ± 0.06	0.984 ± 0.05	0.778	0.993 ± 0.06	0.978 ± 0.05	0.590	0.034
Muscle mass, kg	33.0 ± 7.7	32.1 ± 7.1	0.677	31.2 ± 7.3	30.1 ± 6.6	0.967	0.315
Fat mass, kg	46.7 ± 10.3	42.7 ± 9.8	0.169	46.4 ± 7.9	42.4 ± 9.6	0.211	0.180
Total water content, L	42.6 ± 10.2	41.2 ± 9.7	0.001	41.1 ± 8.8	40.7 ± 7.8	0.072	0.252
Morning SBP, mmHg	134.0 ± 1.6	121.8 ± 1.5	<0.001	131.4 ± 1.8	120.0 ± 1.8	<0.001	0.978
Evening SBP, mmHg	129.4 ± 2.5	120.6 ± 1.2	<0.001	130.0 ± 3.3	119.3 ± 1.6	0.004	0.716
Morning DBP, mmHg	82.4 ± 1.2	78.4 ± 0.9	0.040	82.1 ± 1.5	78.6 ± 1.0	0.002	0.240
Evening DBP, mmHg	79.6 ± 1.4	78.0 ± 1.2	0.528	79.6 ± 1.6	76.4 ± 1.3	0.008	0.026

Data are presented as mean ± standard deviation. BMI, body mass index; DBP, diastolic blood pressure;

SBP, systolic blood pressure.

**Table 5:** Urinarypolyamines (mmol/mol of creatinine) in probiotic and control groups

**Laboratory measurement**	**Probiotic**			**Control**			**Extent of change**
	**Baseline**	**After treatment**	***P***	**Baseline**	**After treatment**	***P***	**Probiotic vs. control food** ***P***
Creatinine	14462 ± 8075	12431 ± 6152	0.322	10512 *±* 6509	12977 ± 6381	0.967	0.262
Putrescine	0.117 ± 0.07	0.166 ± 0.14	0.207	0.161 ± 0.09	0.110 ± 0.07	0.019	0.014
Acetylated putrescine	0.291 ± 0.33	0.524 ± 1.36	0.410	0.434 *±* 0.36	0.192 ± 0.11	0.004	0.036
Tyramine	0.041 ± 0.12	0.0098 ± 0.05	NS	0.074 *±* 0.20	0		
Acetylatedspermidine	0.220 ± 0.296	0.601 ± 1.85	0.277	0.202 *±* 0.12	0.183 *±* 0.11	0.115	0.157

Note: 1.3-diaminopropane, cadaverine, histamine, and spermidine were not detected.

**Table 6:** Spearman’s partial correlation analysis of probiotic and control groups together (n = 36) for studied variables adjusted for study group, baseline BMI, age, and sex

**Associated variables**		**Coefficient r**	**p**
BMI	Water content**	0.570	0.0007
	Lactobacilli content *	0.383	0.0305
Lactobacilli content	Putrescine content*	0.475	0.0060
Colonization with TENSIA	Morning SBP*	−0.527	0.0640
	Morning DBP**	0.617	0.0248
Acetylated spermidine	Morning DBP**	r = −0.417	p = 0.0177
	Putrescine**	r = 0.714	p < 0.0001
	Water content*	r = −0.361	p = 0.0426

BMI, body mass index; DBP, diastolic blood pressure; SBP, systolic blood pressure.

*Values at the end of the study.

**Differences between the extent of change (final values - initial values).

TENSIA subgroup analysis (n = 16) for correlations between TENSIA colonization and other studied variables.

## Results

The anthropometric and clinical indices of the two participant groups did not differ. The number of patients taking medical drugs was also similar between the two groups (84% of the probiotic group vs. 80% of the control group), and the distribution of drugs did not differ between the two groups (Table 2). The consumption of probiotic cheese did not cause any adverse effects aside from some temporary constipation, which occurred at similar rates in the two groups. The extents of change in body weight (−5.7 vs.-4.4 kg, p = 0.083) and BMI (−2 vs. -1.6 kg/m^2^, p = 0.031) were larger in the probiotic group than the control group at the end of the study (Table 4). Concerning the body composition, a decrease of the total water content (p = 0.001) was found only in the probiotic group, however, the data of water, fat and muscle mass and waist-to-hip ratio did not differ between probiotic and control groups. All patients treated with BP-lowering drugs registered a similar significant reduction of both SBP and diastolic blood pressure (DBP), regardless of the type of cheese ingested (Table 4).

Although the consumption of full-fat probiotic cheese reduced the values of total cholesterol, low-density lipoprotein, high-density lipoprotein, and triglycerides, no differences in the extents of change were observed between the probiotic and control groups (Table 3). Triglycerides (p = 0.041) were reduced only in probiotic cheese consumers. Although an 18% reduction of blood glucose level was observed in both groups, statistical significance was reached only in the control group A significant increase of urea content was registered in consumers of probiotic cheese (p = 0.011), although the urea level remained within the normal range. No differences in the extents of change in probiotic and control groups were observed regarding uric acid, total bilirubin, AST or ALT levels.

Urinary putrescine and its derivative, acetylated putrescine, exhibited significant change in probiotic versus control groups, while a significant reduction of these polyamines was detected in the control group. No changes were detected in tyramine or acetylated spermidine (Table 5).

**Figure 2:**
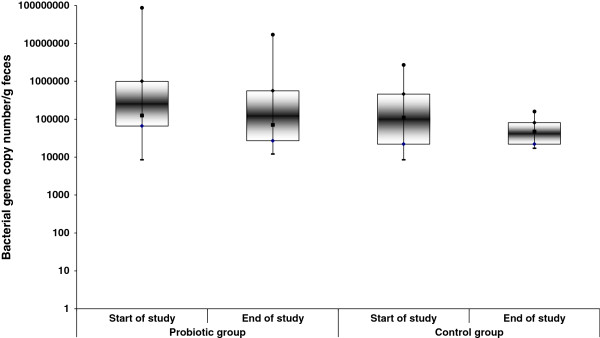
**Changes in the gene copy numbers of lactobacilli in feces of probiotic and control groups.** The gene copy numbers of total lactobacilli per gram of feces were determined usingreal-time PCR assay with specific primers. The counts of total lactobacilli decreased in the control group (n = 15, P = 0.056), not in the probiotic group (n = 25*,* P = 0.497*).* Dot plots indicate max-min, median, and 1st and 3rd quartiles.

The total count of lactobacilli (Figure 2) did not change after the consumption of probiotic cheese; however, a large range was observed among individuals at the beginning of the study. In contrast, in the control group, there was a trend towards lower total lactobacilli counts (p = 0.056). The probiotic *L. plantarum* TENSIA was observed in 16/25 (64%) probiotic-treated subjects in varying amounts (529.6 ± 232.5 gene copies).

### Comparison of colonization with lactobacilli and *L. plantarum* TENSIA, and correlation of colonization with BMI, water content of body, arterial blood pressure, and urinary polyamines

Spearman’s partial correlation analyses for both control and TENSIA groups combined was applied to 36 individuals because the urine samples were missing in 4 controls. It was detected that the extents of change in water content significantly affected the changes in BMI (r = 570, p = 0.0014) when adjusted for sex and age (Table 6). The data of TENSIA subgroup alone (n = 16 participants who were colonized) detected that the higher lactobacilli counts at the end of the study were positively associated with higher BMI (r = 0.383 p = 0.0305) and higher putrescine level (r = 0.475 p = 0.006). At the same time, the changes in urinary putrescine and acetylated spermidine during the study were tightly inter-connected (r = 0.714, p = 0.0001; Table 6). The magnitude of change of acetylated spermidine was negatively associated with changes of DBP at morning measurements (r = −0.417 p = 0.0445) and water content of body (r = −0.361, p = 0.0426). After adjustment for BMI, age, and sex, a positive association was observed between TENSIA colonization and the extent of change of DBP at morning (r = 0.617 p = 0.0248); a trend toward lower morning SBP (r = −0.527 p = 0.0640) was also observed.

## Discussion

Implementation of a hypocaloric diet supplemented with probiotic cheese containing *L. plantarum* TENSIA, in a double-blind, randomized pilot study resulted in several beneficial shifts of health markers in overweight and obese subjects. First, the consumption of probiotic cheese was associated with more efficient reduction of BMI compared with ordinary cheese. This effect was correlated with a significant decrease of water content after probiotic cheese consumption, adjusted for sex and age. Among patients who were taking BP-lowering drugs, there was a significant association between *L. plantarum* TENSIA colonization and both SBP and DBP at the end of the study, as well as the magnitude of the observed changes in each. In both the probiotic and control groups, the reduction of total cholesterol and low-density lipoprotein was observed after the consumption of 50 g of cheese containing 26% fat for 3 weeks. Significant reduction of the plasma triglyceride level was detected only in the probiotic group.

The use of probiotics to reduce the body weight and cholesterol and triglyceride indices of obese patients seems challenging [27,28]. However, several clinical and experimental studies have shown that probiotic lactobacilli belonging to the genus *Firmicutes* did not help to reduce BMI [29,30]. Species differences may be responsible for this finding, yet. For instance, a report indicates that higher BMI is significantly related to homofermentative *L. acidophilus* in an age- and sex-adjusted population of healthy volunteers (29).

*L. plantarum* is a heterofermentative lactic acid bacterium that has been found in a large range of environmental niches [31-35]. It has a proven ability to survive gastric and intestinal transit and easily colonizes the human intestinal tract. Functional properties of *L. plantarum* TENSIA include antimicrobial activity; production of polyamines and nitric oxide; and moderate anti-oxidative ability [20]. Several studies by different investigators have described the beneficial effects of the consumption of the species *L. plantarum* on human physiology [31,36,37].

Although Smith et al. postulated that responsiveness to probiotics may vary among individuals; such differences do not persist when probiotics are consumed for longer than 9 days [38]. In our study even after 3 weeks of consumption, the consumed strain was detectable in only 64% of patients, and there were quite large differences in the numbers of gene copies of strain TENSIA. This range of gene copies may have affected the total counts of lactobacilli, the magnitude of change in BP indices at the end of the study, and the metabolic data tested.

Plausible mechanisms to explain the associations between health indices and dairy intake are still lacking. A Japanese multicenter study of 87 overweight people (BMI of 24.2 kg/m^2^) who consumed 200 g of fermented milk per day with or without *Lactobacillus gasseri* SBT2055 for 12 weeks demonstrated a 1.5% reduction in BMI and hip circumference in the intervention group and no reductions in the control group [28]. In the present study, we observed a similar reduction of BMI after just 3 weeks of consuming probiotic cheese accompanied with some other beneficial changes. Similar to the findings of McNulty et al. [39], we did not observe an increase of the total number of fecal lactobacilli. However, in our study the presence and DNA copy number of TENSIA were associated with BP reduction. The shifts of lactobacilli count after probiotic intervention that influenced the host metabolism might have been detected in upper parts of intestine [29,40].

In the present pilot study, the reduction of arterial BP was mainly achieved by BP-lowering medications; however, there was a relevant trend toward an association between the lowering of both morning SBP and DBP and TENSIA colonization. The reduction in SBP and DBP after 8 weeks of intervention has been shown in mildly hypertensive subjects using *Lactobacillus helveticus* or *Saccharomyces cerevisiae*[41,42]. During casein degradation by *Lactobacillus helveticus,* angiotensin I-converting enzyme (ACE) inhibitory peptides were produced that exerted an antihypertensive effect *in vivo*[43]. ACE is a highly selective ecto-enzyme that is involved in the regulation of peripheral BP [44]. We detected the ACE inhibitory activity of *L. plantarum* TENSIA in preliminary *in vitro* studies (unpublished data). In addition, some other lactobacilli, such as *Lactobacillus animalis,* produce some bioactive fractions from caseins that possess ACE-inhibitory activities [45].

The mechanisms behind the arterial BP-lowering effect of our study may include vascular relaxation, which is also correlated with the reduction of excess reactive oxygen species in vascular biology [46,47]. Our strain, *L. plantarum* TENSIA, has moderate antioxidant activity and is able to decarboxylate ornithine and produce detectable putrescine in urine [20]. Large amounts of endogenous and exogenous polyamines are present in the gut lumen of healthy humans and stimulate DNA, RNA, and protein synthesis [48,49] and the maturation of large intestinal mucosa [50,51]. Although a strong association between exposure to increased counts of intestinal lactobacilli and increased urinary putrescine was detected in our patients, the amount of excreted polyamines did not change significantly at the end of the intervention. However, the more pronounced changes of acetylated spermidine in urine were associated with lower changes of DBP when measured in the morning and with lower content of water in the body. The action of polyamines, including their hypotensive effects, appears to depend closely on their serum concentration [52]. In addition, the production of nitric oxide by the TENSIA strain *in vitro*[20] also hints at the relaxation of blood vessels; this may be the mechanism linking consumption of TENSIA with BP lowering.

A randomized controlled trial has demonstrated the effects of both milk and soy proteins on lowering BP compared with a carbohydrate-rich diet [53]. In the present human trial, the impact of *L. plantarum* TENSIA on protein catabolism was demonstrated by the increase of urea in blood. Increased serum urea values are usually caused by high protein diets and/or with some starvation. It is possible that higher counts of lactobacilli in patients consuming cheese with *L. plantarum* Tensia caused the decrease of the pH of gut. This could be accompanied with some increase of protein putrefaction and blood urea content previously described in experimental animals and metabolic surgery [54-56]. However, in our patients the increased urea values were still kept in normal ranges and also no shifts in uric acid were detected relevant for excess of protein catabolism towards health impairment. Moreover, the functionality of kidneys and liver was not altered by the 3 weeks treatment with hypocaloric diet and probiotic cheese. The possibility to prevent water retention and hypertension with the hypocaloric diet supplemented with probiotic cheese seems worth larger clinical and translational studies.

The important findings of this study were that the consumption of a hypocaloric diet supplemented with protein-rich full-fat cheese resulted in the lowering of blood glucose levels by 18% in both study and control groups, while no increase in total cholesterol, low-density lipoprotein, or triglycerides were observed.

Thus, there is good potential for probiotic cheese containing *L. plantarum* TENSIA to be included in a hypocaloric diet to reduce the symptoms of metabolic syndrome. This finding may broaden the area of non-medication methods that can be employed to achieve optimal arterial BP values and normalization of the BMI, which currently includes healthy nutrition, quitting smoking, and increasing regular physical activity.

In conclusion, the hypocaloric diet supplemented with a probiotic cheese can help reduce BMI, arterial BP, and the risk of metabolic syndrome in obese patients with hypertension.

## Abbreviations

ACE: Angiotensin I-converting enzyme; ALT: Alanine aminotransferase; AST: Aspartate aminotransferase; BMI: Body mass index; BP: Blood pressure; DBP: Diastolic blood pressure; NORIP: Nordic Reference Interval Project; RT-PCR: Real-time polymerase chain reaction; SBP: Systolic blood pressure.

## Competing interests

MM and ES are among the authors of the probiotic *Lactobacillus plantarum* TENSIA patent. None of the other authors have any conflicts of interest to report.

## Authors’ contributions

SKK, ES, and MM designed the trial; POA, ARI, STB, and KBS conducted the clinical study; JS and IS performed the analysis of human bio-specimens; SKK, ES, and MM analyzed the data and wrote the manuscript; MM had primary responsibility for the final content. All authors read and approved the final manuscript.
